# Absolute vs. relative effects—implications for subgroup analyses

**DOI:** 10.1186/s13063-020-05005-7

**Published:** 2021-01-11

**Authors:** Lars W. Andersen

**Affiliations:** Aarhus University and Aarhus University Hospital, Palle Juul-Jensens Blvd. 99, 8200 Aarhus, Denmark

Subgroup analyses assess whether a given effect measure differs according to baseline characteristics [1]. In general, treatment effects can be measured on a relative (i.e., odds ratio or risk ratio) or absolute (i.e., risk difference) scale when an outcome is binary. If a treatment has an effect and the control-group outcome differs according to the subgroup characteristic, conclusions about subgroup differences will depend on the scale of the effect measure used.

To evaluate the reporting of subgroup analyses in the *NEJM*, a review of the last 100 randomized trials reporting subgroup analyses was performed (see Supplemental Appendix for methodological details). Twenty-eight trials reported a binary primary outcome. The primary results were reported as an odds ratio in four trials (14%), risk ratio in 13 trials (47%), and a risk difference in 11 trials (39%). Five trials (18%) reported two effects measures. There was substantial heterogeneity in the effect measure used for subgroup analyses: 17 trials (61%) assessed subgroup differences on a relative scale, nine trials (32%) assessed subgroup differences on an absolute scale, one trial (4%) reported both scales, and one trial (4%) did not report the scale.

To illustrate how the scale can have importance for interpretation of results, subgroup analyses according to estimated baseline risk (low, intermediate, high) were performed using data from SPRINT [2] and PARAMEDIC2 [3] (see Supplemental Appendix for methodological details). Relatively constant risk ratios across baseline risks in SPRINT translated to more variability in risk differences (Fig. 1, Table S1). By contrast, substantial differences in risk ratios across baseline risk in PARAMEDIC2 translated to more constant (although still different) risk differences (Fig. 1, Table S1).

**Fig. 1:**
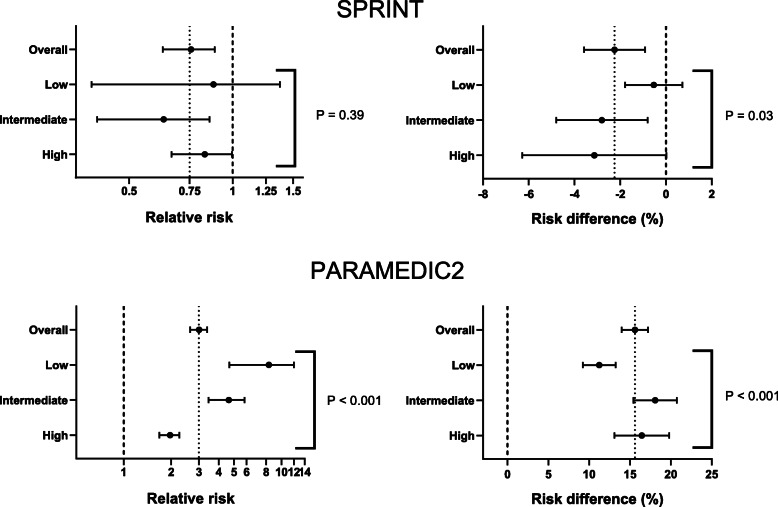
Subgroup analyses based on estimated baseline risk. The outcome of interest was the primary outcome from SPRINT (a composite of cardiovascular outcomes) and survival to hospital admission for PARAMEDIC2. Patients were divided into three groups based on their baseline risk of the outcome (low, intermediate, high). Subgroup analyses were then performed within these categories on the relative scale (left) and the absolute scale (right). The thin dashed vertical line represents the effect in the overall cohort. The thick dashed vertical line represents no effect. The *P* value is from the subgroup and intervention interaction. Additional details are provided in the Supplemental Appendix

Meta-epidemiological studies suggest that relative effects are generally more constant across baseline risk as compared to absolute effects [4–6]. Some authors therefore argue that subgroup analyses on the relative scale are of most interest [7]. However, as demonstrated here, subgroup analyses do not consistently abide by this rule. Despite recommendations to present absolute risk differences in addition to relative measures when reporting clinical trials [8], the findings of this study suggest it is not common practice for subgroup analyses. As relative and absolute risks are often interpreted differently, the choice of scale could have implications for behavior and treatment choices. Absolute risk differences are particularly relevant for decision making and public health, as effect measure modification on the absolute scale is believed to represent actual biological interaction between the subgroup characteristic and the intervention [9]. Given the implications for interpretation, authors should consider reporting subgroup analyses on both the absolute and the relative scale or, as a minimum, justify the scale used.

## Supplementary Information

**Additional file 1.**

## Data Availability

Data from SPRINT can be requested through the National Heart, Lung, and Blood Institute. Data from PARAMEDIC2 can be requested from Gavin Perkins, M.D. and the Warwick Clinical Trials Unit at the University of Warwick. Data obtained through the review of trials can be requested from the author.
