# Supplementary material for: Absolute vs. relative effects—implications for subgroup analyses
Source: Trials. 2021 Jan 11;22:50. doi: 10.1186/s13063-020-05005-7 (PMC7802340; doi:10.1186/s13063-020-05005-7)
Supplement: Supplementary file 1 — Additional file 1. [file 13063_2020_5005_MOESM1_ESM.docx]

**Supplemental Appendix**

Absolute vs. Relative Effects – Implications for Subgroup Analyses

**Systematic review**

To evaluate reporting of subgroup results in the *NEJM*, a systematic review was performed of 100 recent randomized clinical trials. The review included all trials reporting subgroup analysis. Trials using non-frequentist statistics (e.g. Bayesian statistics), trials assessing vaccine efficacy, non-inferiority trials, and cluster-randomized trials were excluded.

The following search strategy was used on PubMed on March 17^th^, 2020:

*NEJM[jour] AND (randomized controlled trial[pt] OR controlled clinical trial[pt] OR randomized[tiab] OR placebo[tiab] OR drug therapy[sh] OR randomly[tiab] OR trial[tiab] OR groups[tiab] NOT (animals [mh] NOT humans [mh]))*

One reviewer assessed the identified manuscripts for eligibility. The 100 newest trials were included.[1-100] These were published between December 2018 and February 2020. The majority of trials were within oncology (29%) or cardiology (22%). Most trials (73%) tested pharmacological interventions.

**Subgroup analyses**

*Choice of data set and data sharing*

To illustrate the potential discrepancy between subgroup analyses on the absolute and relative scale, analyses were done on two recent large clinical trials published in the *NEJM*, namely SPRINT and PARAMEDIC2. SPRINT compared intensive blood pressure lowering (< 120 mmHg systolic) to standard of care (< 140 mmHg systolic) in patients with an increased cardiovascular risk.[101] PARAMEDIC2 compared epinephrine to placebo for patients with out-of-hospital cardiac arrest.[102]

These trials were chosen for a number of reasons: 1) The trials were large (including 9361 and 8014 patients, respectively) allowing for meaningful subgroup analyses, 2) both trials showed a statistically significant difference in outcomes, and 3) data were available for sharing.

Deidentified SPRINT data were obtained through BioLINCC at the National Heart, Lung, and Blood Institute. Deidentified PARAMEDIC2 data were obtained through Gavin Perkins, M.D. and the Warwick Clinical Trials Unit at University of Warwick.

Access to deidentified data of this type does not require ethical approval in Denmark.

*SPRINT*

For the current analysis, patients were included based on the inclusion/exclusion criteria used in the original primary analysis.[101] For simplicity, patients with missing baseline data or no 3-year outcome data were excluded. This allowed for the 3-year outcome to be treated as a binary outcome. The outcome of interest was a composite of myocardial infarction, acute coronary syndrome not resulting in myocardial infarction, stroke, acute decompensated heart failure, or death from cardiovascular causes at 3 years. Baseline variables included age, sex, race/ethnic group, baseline systolic and diastolic blood pressure, estimated glomerular filtration rate, fasting total cholesterol, HDL cholesterol, total triglycerides, and plasma glucose, statin use, aspirin use, number of antihypertensive agents, smoking, body mass index, and the Framingham 10-year risk score.

*PARAMEDIC2*

For the current analysis, patients were included based on the inclusion/exclusion criteria used in the original primary analysis.[103] For simplicity, patients with missing baseline data or missing data on survival to hospital admission were excluded. The outcome of interest was survival to hospital admission. This outcome was chosen over the primary outcome used in the original trial (i.e. survival to hospital discharge) because of the large effect size allowing for more meaningful subgroup analyses. Baseline variables included age, sex, location of the cardiac arrest, presumed cardiac arrest etiology, initial rhythm, witnessed status, receipt of bystander cardiopulmonary resuscitation, and time from emergency call to ambulance arrival at the scene.

*Defining baseline risk*

For both cohorts, a prediction model was created to estimate the probability that a patient would have the outcome of interest. These estimates were obtained from a logistic regression model with the relevant outcome as the dependent variable and the above-mentioned baseline variables as the independent variables. All continuous variables were categorized into deciles. Based on the estimated probabilities, each cohort was then divided into tertiles based on baseline risk of the outcome; low, intermediate, or high.

*Estimation of risk ratios and risks differences*

Risk ratios and risk differences for the outcomes were estimated comparing the intervention to the control. Estimates were obtained both for the entire cohort and within the created subgroups based on baseline risk. Risk ratios were obtained from log-binomial models (log link function, binomial distribution) and risk difference from linear models (identity link function, binomial distribution), both within the framework of Generalized Linear Models.[104] Effect measure modification was assessed on both scales based on the P value from the interaction between the intervention and subgroup.

*Model performance*

Performance, in terms of discrimination and calibration, of the baseline risk models is illustrated in Figure S1. Both models had moderate discrimination ability and excellent calibration.

**Figure and Table**

**Figure S1. Performance of the baseline risk models**

Results from the SPRINT and PARAMEDIC2 baseline risk models are illustrated with predicted vs. actual outcome plots for each subgroup (left) and received operating characteristics (ROC) curves on the right.

*AUC indicates area under the curve.*

| **Table S1. Risk differences (RD) and risk ratios (RR)** | | | | |
| --- | --- | --- | --- | --- |
|  | **Intervention** | **Comparison** | **RD - %**  **(95%CI)** | **RR**  **(95%CI)** |
| **SPRINT** | | | | |
| **Overall** | 211/3121  (6.8%) | 281/3120  (9.0%) | -2.3  (-3.6, -0.1) | 0.75  (0.63, 0.89) |
| **Low risk** | 20/1032  (1.9%) | 25/1014  (2.5%) | -0.5  (-1.8, 0.7) | 0.79  (0.44, 1.41) |
| **Intermediate risk** | 44/1037  (4.2%) | 71/1010  (7.0%) | -2.8  (-4.8, -0.8) | 0.60  (0.42, 0.87) |
| **High risk** | 143/1007  (14.2%) | 180/1040  (17.3%) | -3.1  (- 6.3, 0.0) | 0.82  (0.67, 1.00) |
| **PARAMEDIC2** | | | | |
| **Overall** | 874/3730  (23.4%) | 294/3748  (7.8%) | 15.6  (14.0, 17.2) | 2.99  (2.64, 3.38) |
| **Low risk** | 157/1219  (12.9%) | 21/1270  (1.7%) | 11.2  (9.2, 13.2) | 7.79  (4.97, 12.2) |
| **Intermediate risk** | 284/1225  (23.2%) | 65/1271  (5.1%) | 18.1  (15.4, 20.7) | 4.53  (3.50, 5.87) |
| **High risk** | 433/1286  (33.7) | 208/1207  (17.2%) | 16.4  (13.1, 19.8) | 1.95  (1.69, 2.26) |

**References**

1. Schmid, P., et al., *Pembrolizumab for Early Triple-Negative Breast Cancer.* N Engl J Med, 2020. **382**(9): p. 810-821.

2. Walsh, M., et al., *Plasma Exchange and Glucocorticoids in Severe ANCA-Associated Vasculitis.* N Engl J Med, 2020. **382**(7): p. 622-631.

3. de Koning, H.J., et al., *Reduced Lung-Cancer Mortality with Volume CT Screening in a Randomized Trial.* N Engl J Med, 2020. **382**(6): p. 503-513.

4. Makkar, R.R., et al., *Five-Year Outcomes of Transcatheter or Surgical Aortic-Valve Replacement.* N Engl J Med, 2020. **382**(9): p. 799-809.

5. Juul, S.E., et al., *A Randomized Trial of Erythropoietin for Neuroprotection in Preterm Infants.* N Engl J Med, 2020. **382**(3): p. 233-243.

6. Finkelstein, A., et al., *Health Care Hotspotting - A Randomized, Controlled Trial.* N Engl J Med, 2020. **382**(2): p. 152-162.

7. Fenaux, P., et al., *Luspatercept in Patients with Lower-Risk Myelodysplastic Syndromes.* N Engl J Med, 2020. **382**(2): p. 140-151.

8. Voskoboinik, A., et al., *Alcohol Abstinence in Drinkers with Atrial Fibrillation.* N Engl J Med, 2020. **382**(1): p. 20-28.

9. Ray-Coquard, I., et al., *Olaparib plus Bevacizumab as First-Line Maintenance in Ovarian Cancer.* N Engl J Med, 2019. **381**(25): p. 2416-2428.

10. Morand, E.F., et al., *Trial of Anifrolumab in Active Systemic Lupus Erythematosus.* N Engl J Med, 2020. **382**(3): p. 211-221.

11. Slamon, D.J., et al., *Overall Survival with Ribociclib plus Fulvestrant in Advanced Breast Cancer.* N Engl J Med, 2020. **382**(6): p. 514-524.

12. National Heart, L., et al., *Early High-Dose Vitamin D3 for Critically Ill, Vitamin D-Deficient Patients.* N Engl J Med, 2019. **381**(26): p. 2529-2540.

13. Murthy, R.K., et al., *Tucatinib, Trastuzumab, and Capecitabine for HER2-Positive Metastatic Breast Cancer.* N Engl J Med, 2020. **382**(7): p. 597-609.

14. Yamamura, T., et al., *Trial of Satralizumab in Neuromyelitis Optica Spectrum Disorder.* N Engl J Med, 2019. **381**(22): p. 2114-2124.

15. Mulangu, S., et al., *A Randomized, Controlled Trial of Ebola Virus Disease Therapeutics.* N Engl J Med, 2019. **381**(24): p. 2293-2303.

16. Ramalingam, S.S., et al., *Overall Survival with Osimertinib in Untreated, EGFR-Mutated Advanced NSCLC.* N Engl J Med, 2020. **382**(1): p. 41-50.

17. Amarenco, P., et al., *A Comparison of Two LDL Cholesterol Targets after Ischemic Stroke.* N Engl J Med, 2020. **382**(1): p. 9.

18. Kang, D.H., et al., *Early Surgery or Conservative Care for Asymptomatic Aortic Stenosis.* N Engl J Med, 2020. **382**(2): p. 111-119.

19. Dangas, G.D., et al., *A Controlled Trial of Rivaroxaban after Transcatheter Aortic-Valve Replacement.* N Engl J Med, 2020. **382**(2): p. 120-129.

20. Tardif, J.C., et al., *Efficacy and Safety of Low-Dose Colchicine after Myocardial Infarction.* N Engl J Med, 2019. **381**(26): p. 2497-2505.

21. Coleman, R.L., et al., *Secondary Surgical Cytoreduction for Recurrent Ovarian Cancer.* N Engl J Med, 2019. **381**(20): p. 1929-1939.

22. Hatemi, G., et al., *Trial of Apremilast for Oral Ulcers in Behcet's Syndrome.* N Engl J Med, 2019. **381**(20): p. 1918-1928.

23. Fishbane, S., et al., *A Phase 3 Trial of Difelikefalin in Hemodialysis Patients with Pruritus.* N Engl J Med, 2020. **382**(3): p. 222-232.

24. Middleton, P.G., et al., *Elexacaftor-Tezacaftor-Ivacaftor for Cystic Fibrosis with a Single Phe508del Allele.* N Engl J Med, 2019. **381**(19): p. 1809-1819.

25. Francois, B., et al., *Prevention of Early Ventilator-Associated Pneumonia after Cardiac Arrest.* N Engl J Med, 2019. **381**(19): p. 1831-1842.

26. Perl, A.E., et al., *Gilteritinib or Chemotherapy for Relapsed or Refractory FLT3-Mutated AML.* N Engl J Med, 2019. **381**(18): p. 1728-1740.

27. Dransfield, M.T., et al., *Metoprolol for the Prevention of Acute Exacerbations of COPD.* N Engl J Med, 2019. **381**(24): p. 2304-2314.

28. Investigators, I.-R., et al., *Conservative Oxygen Therapy during Mechanical Ventilation in the ICU.* N Engl J Med, 2020. **382**(11): p. 989-998.

29. Dorling, J., et al., *Controlled Trial of Two Incremental Milk-Feeding Rates in Preterm Infants.* N Engl J Med, 2019. **381**(15): p. 1434-1443.

30. Lascarrou, J.B., et al., *Targeted Temperature Management for Cardiac Arrest with Nonshockable Rhythm.* N Engl J Med, 2019. **381**(24): p. 2327-2337.

31. de Wit, R., et al., *Cabazitaxel versus Abiraterone or Enzalutamide in Metastatic Prostate Cancer.* N Engl J Med, 2019. **381**(26): p. 2506-2518.

32. Kopetz, S., et al., *Encorafenib, Binimetinib, and Cetuximab in BRAF V600E-Mutated Colorectal Cancer.* N Engl J Med, 2019. **381**(17): p. 1632-1643.

33. Flaherty, K.R., et al., *Nintedanib in Progressive Fibrosing Interstitial Lung Diseases.* N Engl J Med, 2019. **381**(18): p. 1718-1727.

34. Coleman, R.L., et al., *Veliparib with First-Line Chemotherapy and as Maintenance Therapy in Ovarian Cancer.* N Engl J Med, 2019. **381**(25): p. 2403-2415.

35. Gonzalez-Martin, A., et al., *Niraparib in Patients with Newly Diagnosed Advanced Ovarian Cancer.* N Engl J Med, 2019. **381**(25): p. 2391-2402.

36. Stone, G.W., et al., *Five-Year Outcomes after PCI or CABG for Left Main Coronary Disease.* N Engl J Med, 2019. **381**(19): p. 1820-1830.

37. Larkin, J., et al., *Five-Year Survival with Combined Nivolumab and Ipilimumab in Advanced Melanoma.* N Engl J Med, 2019. **381**(16): p. 1535-1546.

38. Hellmann, M.D., et al., *Nivolumab plus Ipilimumab in Advanced Non-Small-Cell Lung Cancer.* N Engl J Med, 2019. **381**(21): p. 2020-2031.

39. Investigators, H., et al., *Total Hip Arthroplasty or Hemiarthroplasty for Hip Fracture.* N Engl J Med, 2019. **381**(23): p. 2199-2208.

40. Mehran, R., et al., *Ticagrelor with or without Aspirin in High-Risk Patients after PCI.* N Engl J Med, 2019. **381**(21): p. 2032-2042.

41. Sands, B.E., et al., *Vedolizumab versus Adalimumab for Moderate-to-Severe Ulcerative Colitis.* N Engl J Med, 2019. **381**(13): p. 1215-1226.

42. Sands, B.E., et al., *Ustekinumab as Induction and Maintenance Therapy for Ulcerative Colitis.* N Engl J Med, 2019. **381**(13): p. 1201-1214.

43. McMurray, J.J.V., et al., *Dapagliflozin in Patients with Heart Failure and Reduced Ejection Fraction.* N Engl J Med, 2019. **381**(21): p. 1995-2008.

44. Maertens, J., et al., *Maribavir for Preemptive Treatment of Cytomegalovirus Reactivation.* N Engl J Med, 2019. **381**(12): p. 1136-1147.

45. Munoz, D., et al., *Polypill for Cardiovascular Disease Prevention in an Underserved Population.* N Engl J Med, 2019. **381**(12): p. 1114-1123.

46. Makrides, M., et al., *A Randomized Trial of Prenatal n-3 Fatty Acid Supplementation and Preterm Delivery.* N Engl J Med, 2019. **381**(11): p. 1035-1045.

47. Tazawa, R., et al., *Inhaled GM-CSF for Pulmonary Alveolar Proteinosis.* N Engl J Med, 2019. **381**(10): p. 923-932.

48. Schupke, S., et al., *Ticagrelor or Prasugrel in Patients with Acute Coronary Syndromes.* N Engl J Med, 2019. **381**(16): p. 1524-1534.

49. Steg, P.G., et al., *Ticagrelor in Patients with Stable Coronary Disease and Diabetes.* N Engl J Med, 2019. **381**(14): p. 1309-1320.

50. Mehta, S.R., et al., *Complete Revascularization with Multivessel PCI for Myocardial Infarction.* N Engl J Med, 2019. **381**(15): p. 1411-1421.

51. Solomon, S.D., et al., *Angiotensin-Neprilysin Inhibition in Heart Failure with Preserved Ejection Fraction.* N Engl J Med, 2019. **381**(17): p. 1609-1620.

52. Metra, M., et al., *Effects of Serelaxin in Patients with Acute Heart Failure.* N Engl J Med, 2019. **381**(8): p. 716-726.

53. Witztum, J.L., et al., *Volanesorsen and Triglyceride Levels in Familial Chylomicronemia Syndrome.* N Engl J Med, 2019. **381**(6): p. 531-542.

54. Shanafelt, T.D., et al., *Ibrutinib-Rituximab or Chemoimmunotherapy for Chronic Lymphocytic Leukemia.* N Engl J Med, 2019. **381**(5): p. 432-443.

55. Maitland, K., et al., *Transfusion Volume for Children with Severe Anemia in Africa.* N Engl J Med, 2019. **381**(5): p. 420-431.

56. Maitland, K., et al., *Immediate Transfusion in African Children with Uncomplicated Severe Anemia.* N Engl J Med, 2019. **381**(5): p. 407-419.

57. Vichinsky, E., et al., *A Phase 3 Randomized Trial of Voxelotor in Sickle Cell Disease.* N Engl J Med, 2019. **381**(6): p. 509-519.

58. Matei, D., et al., *Adjuvant Chemotherapy plus Radiation for Locally Advanced Endometrial Cancer.* N Engl J Med, 2019. **380**(24): p. 2317-2326.

59. Herold, K.C., et al., *An Anti-CD3 Antibody, Teplizumab, in Relatives at Risk for Type 1 Diabetes.* N Engl J Med, 2019. **381**(7): p. 603-613.

60. Pittas, A.G., et al., *Vitamin D Supplementation and Prevention of Type 2 Diabetes.* N Engl J Med, 2019. **381**(6): p. 520-530.

61. Fischer, K., et al., *Venetoclax and Obinutuzumab in Patients with CLL and Coexisting Conditions.* N Engl J Med, 2019. **380**(23): p. 2225-2236.

62. Im, S.A., et al., *Overall Survival with Ribociclib plus Endocrine Therapy in Breast Cancer.* N Engl J Med, 2019. **381**(4): p. 307-316.

63. Davis, I.D., et al., *Enzalutamide with Standard First-Line Therapy in Metastatic Prostate Cancer.* N Engl J Med, 2019. **381**(2): p. 121-131.

64. Golan, T., et al., *Maintenance Olaparib for Germline BRCA-Mutated Metastatic Pancreatic Cancer.* N Engl J Med, 2019. **381**(4): p. 317-327.

65. Chi, K.N., et al., *Apalutamide for Metastatic, Castration-Sensitive Prostate Cancer.* N Engl J Med, 2019. **381**(1): p. 13-24.

66. Zhang, Y., et al., *Gemcitabine and Cisplatin Induction Chemotherapy in Nasopharyngeal Carcinoma.* N Engl J Med, 2019. **381**(12): p. 1124-1135.

67. Lederle, F.A., et al., *Open versus Endovascular Repair of Abdominal Aortic Aneurysm.* N Engl J Med, 2019. **380**(22): p. 2126-2135.

68. Facon, T., et al., *Daratumumab plus Lenalidomide and Dexamethasone for Untreated Myeloma.* N Engl J Med, 2019. **380**(22): p. 2104-2115.

69. Beasley, R., et al., *Controlled Trial of Budesonide-Formoterol as Needed for Mild Asthma.* N Engl J Med, 2019. **380**(21): p. 2020-2030.

70. Lazarus, S.C., et al., *Mometasone or Tiotropium in Mild Asthma with a Low Sputum Eosinophil Level.* N Engl J Med, 2019. **380**(21): p. 2009-2019.

71. National Heart, L., et al., *Early Neuromuscular Blockade in the Acute Respiratory Distress Syndrome.* N Engl J Med, 2019. **380**(21): p. 1997-2008.

72. Shehabi, Y., et al., *Early Sedation with Dexmedetomidine in Critically Ill Patients.* N Engl J Med, 2019. **380**(26): p. 2506-2517.

73. Distler, O., et al., *Nintedanib for Systemic Sclerosis-Associated Interstitial Lung Disease.* N Engl J Med, 2019. **380**(26): p. 2518-2528.

74. Andre, F., et al., *Alpelisib for PIK3CA-Mutated, Hormone Receptor-Positive Advanced Breast Cancer.* N Engl J Med, 2019. **380**(20): p. 1929-1940.

75. Diener, H.C., et al., *Dabigatran for Prevention of Stroke after Embolic Stroke of Undetermined Source.* N Engl J Med, 2019. **380**(20): p. 1906-1917.

76. Coomarasamy, A., et al., *A Randomized Trial of Progesterone in Women with Bleeding in Early Pregnancy.* N Engl J Med, 2019. **380**(19): p. 1815-1824.

77. Ma, H., et al., *Thrombolysis Guided by Perfusion Imaging up to 9 Hours after Onset of Stroke.* N Engl J Med, 2019. **380**(19): p. 1795-1803.

78. Pittock, S.J., et al., *Eculizumab in Aquaporin-4-Positive Neuromyelitis Optica Spectrum Disorder.* N Engl J Med, 2019. **381**(7): p. 614-625.

79. Tamborlane, W.V., et al., *Liraglutide in Children and Adolescents with Type 2 Diabetes.* N Engl J Med, 2019. **381**(7): p. 637-646.

80. Perkovic, V., et al., *Canagliflozin and Renal Outcomes in Type 2 Diabetes and Nephropathy.* N Engl J Med, 2019. **380**(24): p. 2295-2306.

81. Egan, M.F., et al., *Randomized Trial of Verubecestat for Prodromal Alzheimer's Disease.* N Engl J Med, 2019. **380**(15): p. 1408-1420.

82. Mehta, R.S., et al., *Overall Survival with Fulvestrant plus Anastrozole in Metastatic Breast Cancer.* N Engl J Med, 2019. **380**(13): p. 1226-1234.

83. Dhillon-Smith, R.K., et al., *Levothyroxine in Women with Thyroid Peroxidase Antibodies before Conception.* N Engl J Med, 2019. **380**(14): p. 1316-1325.

84. Landoni, G., et al., *Volatile Anesthetics versus Total Intravenous Anesthesia for Cardiac Surgery.* N Engl J Med, 2019. **380**(13): p. 1214-1225.

85. Lemkes, J.S., et al., *Coronary Angiography after Cardiac Arrest without ST-Segment Elevation.* N Engl J Med, 2019. **380**(15): p. 1397-1407.

86. Tarakji, K.G., et al., *Antibacterial Envelope to Prevent Cardiac Implantable Device Infection.* N Engl J Med, 2019. **380**(20): p. 1895-1905.

87. Lopes, R.D., et al., *Antithrombotic Therapy after Acute Coronary Syndrome or PCI in Atrial Fibrillation.* N Engl J Med, 2019. **380**(16): p. 1509-1524.

88. Mehra, M.R., et al., *A Fully Magnetically Levitated Left Ventricular Assist Device - Final Report.* N Engl J Med, 2019. **380**(17): p. 1618-1627.

89. Ray, K.K., et al., *Safety and Efficacy of Bempedoic Acid to Reduce LDL Cholesterol.* N Engl J Med, 2019. **380**(11): p. 1022-1032.

90. Lissauer, D., et al., *A Randomized Trial of Prophylactic Antibiotics for Miscarriage Surgery.* N Engl J Med, 2019. **380**(11): p. 1012-1021.

91. Harter, P., et al., *A Randomized Trial of Lymphadenectomy in Patients with Advanced Ovarian Neoplasms.* N Engl J Med, 2019. **380**(9): p. 822-832.

92. Khorana, A.A., et al., *Rivaroxaban for Thromboprophylaxis in High-Risk Ambulatory Patients with Cancer.* N Engl J Med, 2019. **380**(8): p. 720-728.

93. Arabi, Y.M., et al., *Adjunctive Intermittent Pneumatic Compression for Venous Thromboprophylaxis.* N Engl J Med, 2019. **380**(14): p. 1305-1315.

94. Rini, B.I., et al., *Pembrolizumab plus Axitinib versus Sunitinib for Advanced Renal-Cell Carcinoma.* N Engl J Med, 2019. **380**(12): p. 1116-1127.

95. Huang, S.S., et al., *Decolonization to Reduce Postdischarge Infection Risk among MRSA Carriers.* N Engl J Med, 2019. **380**(7): p. 638-650.

96. Fizazi, K., et al., *Darolutamide in Nonmetastatic, Castration-Resistant Prostate Cancer.* N Engl J Med, 2019. **380**(13): p. 1235-1246.

97. Taggart, D.P., et al., *Bilateral versus Single Internal-Thoracic-Artery Grafts at 10 Years.* N Engl J Med, 2019. **380**(5): p. 437-446.

98. Lensen, S., et al., *A Randomized Trial of Endometrial Scratching before In Vitro Fertilization.* N Engl J Med, 2019. **380**(4): p. 325-334.

99. Lacerda, M.V.G., et al., *Single-Dose Tafenoquine to Prevent Relapse of Plasmodium vivax Malaria.* N Engl J Med, 2019. **380**(3): p. 215-228.

100. Conroy, T., et al., *FOLFIRINOX or Gemcitabine as Adjuvant Therapy for Pancreatic Cancer.* N Engl J Med, 2018. **379**(25): p. 2395-2406.

101. Sprint Research Group, et al., *A Randomized Trial of Intensive versus Standard Blood-Pressure Control.* N Engl J Med, 2015. **373**(22): p. 2103-16.

102. Perkins, G.D., et al., *A Randomized Trial of Epinephrine in Out-of-Hospital Cardiac Arrest.* N Engl J Med, 2018.

103. Perkins, G.D., et al., *A Randomized Trial of Epinephrine in Out-of-Hospital Cardiac Arrest.* N Engl J Med, 2018. **379**(8): p. 711-721.

104. Naimi, A.I. and B.W. Whitcomb, *Estimating Risk Ratios and Risk Differences Using Regression.* Am J Epidemiol, 2020.
